# Microbiota and immune cell crosstalk: dialogues driving health and disease

**DOI:** 10.1002/cti2.1020

**Published:** 2018-05-28

**Authors:** Mubing Duan

**Affiliations:** ^1^ Department of Biochemistry and Genetics La Trobe Institute for Molecular Science La Trobe University Bundoora VIC 3083 Australia

The therapeutic potential of correcting microbiota dysbiosis, perturbations in the collective composition and functions of bacteria, archaea, fungi and/or virus communities co‐residing within mammalian tissue, has galvanised researchers and clinicians alike. In patients with recurrent *Clostridium difficile* infections, treatment responsiveness to faecal microbiota transplantation (FMT) is approximately 90%.^1^, ^2^ This outcome has not only catapulted an esoteric practice into a choice medical procedure, but also provided solid proof of principle that the transplantation of healthy microbiota can correct dysbiosis and confer biologically advantageous outcomes within a receptive host. Whilst *C. difficile* clearance is particularly amenable to FMT, as the re‐established commensals collectively outcompete *C. difficile* growth, results are modest and heterogeneous for the treatment of inflammatory bowel diseases (IBD).^3^ Attention has therefore been directed towards the rational identification of discrete effectors, whether a minimum combination of microbial species, their molecular products or downstream host cell signalling targets, which can confer the same advantages as a diverse microbiome.

Immune cells, with their co‐evolved capacity to selectively sense and eliminate microbial species, can interact with a local microenvironment yet also migrate into the periphery or distal organs following co‐ordinated activation. This renders them as prime candidates in the endeavour to understand how a localised microbiome may broadly influence organism health and disease susceptibility. Specific commensal microbes can induce tolerogenic or tissue reparative T cells to maintain organ health,^4^, ^5^, ^6^ whilst unintentional microbe translocation can initiate autoantibody and Th17 helper T‐cell (Th17)‐dependent autoimmune disease pathology.^7^ Critically, bidirectional communication exists as B cells produce IgA which can selectively bind to and sequester different microbial species.^8^ Since immune cell contributions to acute and chronic diseases are already extensively studied, knowledge integration of immune cell and microbiota crosstalk may provide new leads in the development of superior therapeutic agents. In this special feature, we present four reviews which address and summarise the evidence for immune cell and microbiota crosstalk during different acute and chronic diseases.

In the review by van den Elsen *et al*.,^9^ commensal microbial maintenance of gastrointestinal integrity and disease‐associated microbiota composition shifts is comprehensively discussed within the context of IBD, metabolic syndrome, allergy and infectious diseases. Diet, which alters the distribution of intestinal microbiota‐accessible carbohydrates, is emphasised to recalibrate host immune and nonimmune cell signalling thresholds towards either tolerogenic or pro‐inflammatory and hence pathological outcomes.

Ubeda *et al*.^10^ further dissect the nonimmune and immune cell‐dependent strategies that intestinal microbiota use to maintain local pathogen resistance. Commensal microbe secretion of bacteriostatic or bactericidal molecules, inhibition of pathogen virulence gene expression and nutrient competition can all deter pathogen colonisation independently of host immunity. However, microbiota induction of immune cell activity, particularly in innate lymphoid cells (ILC), regulatory T cells (Treg) and Th17 cells, is also crucial for the establishment of intestinal pathogen resistance. These interactions are sophisticated, as the co‐ordination of both pro‐inflammatory (Th17 and neutrophil) and tissue protective (Treg and regulatory monocyte) immune cell subsets is required to maximise targeted pathogen killing whilst limiting bystander tissue injury. A degree of functional redundancy also exists between immune cell subsets that secrete the same effector cytokines.

In the review by Shukla *et al*.,^11^ the permutations of gastrointestinal and lung dysbiosis are discussed in the context of asthma, chronic obstructive pulmonary disease (COPD) and cystic fibrosis (CF), three chronic respiratory diseases which contribute substantially to global disease burden. Previously considered as a sterile microenvironment, the lung harbours a dynamic though less dense microbial community compared to the gastrointestinal tract. As either gastrointestinal or lung dysbiosis can be observed during all three respiratory diseases, a communication axis most likely exists whereby gastrointestinal microbiota regulation of lung immunity, or vice versa, occurs through the secretion of inflammatory mediators or migration of activated immune cells. Important disease‐specific research avenues include the study of early life microbiota impact on asthma susceptibility, increasing microbiota resistance against pathogen colonisation to attenuate COPD acute exacerbations and the stratification of microbiota‐dependent variations in CF disease phenotype.^11^

How the microbiota can co‐ordinate complementary changes in seemingly disparate arms of innate and adaptive immunity remains a critical area of investigation. Bauche *et al*.^12^ establish a compelling case for microbiota modulation of growth factor activity as a central axis of immune cell regulation. In the gastrointestinal tract, transforming growth factor‐beta (TGF‐β) maintains tissue integrity through Treg induction, modulation of Th17 pathogenicity and ILC transcriptional identity and increased B‐cell production of IgA. The sum of TGF‐β activity is determined by TGF‐β polypeptide abundance, activation and TGF‐β receptor distribution, and the former two parameters are readily modified by the gastrointestinal microbiota. As other organs require selective growth factors for immune cell homeostasis, notably GM‐CSF in the lungs,^13^, ^14^ further studies into microbiota regulation of growth factor availabilities may uncover new strategies for the modulation of organ‐specific health and disease.

What are the future directions for the study of microbiota and immune cell crosstalk? As the barriers to high‐throughput sequencing technologies decrease, and access to shotgun meta‐omics democratises, precise cataloguing of microbial strain‐specific effects on host cell activity will increase. Whether microbiota‐dependent disease processes co‐opt single or multiple and co‐ordinated immune cell interactions merits investigation, as the minimal conditions required to simulate health‐promoting microbiota remain to be defined. Assays that reorganise microbiota diversity by functional impact may also help uncover new effectors, as well as biomarkers, of specific disease endotypes. As time progresses, one thing is clear that these microscopic interactions will cease to hide under plain sight.
